# Efficacy of BREATHOX^®^ Device Inhalation on Acute Symptoms Associated with COVID-19 (BREATH Study): A Randomized Pilot Clinical Trial

**DOI:** 10.3390/jcm12186075

**Published:** 2023-09-20

**Authors:** Suzana Tanni, Fernando Wehrmeister, Robson Prudente, Felipe Damatto, Carlos Breda Neto, Leiliane Oliveira, Luana Pagan, Mariana Gatto, Letícia Vieira, Liana Coelho, Diane Rezende, Luiz Machado, Gustavo Mota, Marina Gaiato, Felipe Santaella, Elisângela Campos, Estefânia Franco, Matheus Callegari, Marina Politi Okoshi, Ulla Weinreich

**Affiliations:** 1 Medical School, São Paulo State University (Unesp), Distrito de Rubião Junior s/n, Botucatu 18618-970, São Paulo, Brazil; suzanapneumo@gmail.com (S.T.); felipedamatto@hotmail.com (F.D.); ctbredaneto@gmail.com (C.B.N.); lrs.oliveira@unesp.br (L.O.); luanapagan@alunos.fmb.unesp.br (L.P.); marianagatto11@hotmail.com (M.G.); leticiadv@gmail.com (L.V.); lianascoelho@gmail.com (L.C.); dianerezende@gmail.com (D.R.); lh.machado@unesp.br (L.M.); gustavo.mota@unesp.br (G.M.); marina.monte@unesp.br (M.G.); felipe.santaella@unesp.br (F.S.); elisenf21@gmail.com (E.C.); mcallegari10@gmail.com (M.C.); marina.okoshi@unesp.br (M.P.O.); 2 Departament of Social Medicine, The Faculty of Medicine, Federal University of Pelotas, Avenida Duque de Caxias 250, Pelotas 96030-002, Rio Grande do Sul, Brazil; fcwehrmeister@gmail.com; 3 Clinical Hospital of Botucatu Medical School, São Paulo State University (Unesp), Distrito de Rubião Junior s/n, Botucatu 18618-970, São Paulo, Brazil; estefania.franco@unesp.br; 4 Department of Clinical Medicine, The Faculty of Medicine, Aalborg University Hospital, Hobrovej 18-22, 9000 Aalborg, Denmark; ulw@rn.dk

**Keywords:** SARS-CoV-2, hypertonic saline, treatment, mild COVID-19

## Abstract

(1) Background: A high concentration of sodium chloride on in vitro cell culture leads to reduced SARS-CoV-2 replication. Therefore, our aim was to evaluate the effects of inhaling hypertonic NaCl particles (BREATHOX^®^) on the duration of COVID-19-induced acute symptoms. (2) Methods: A prospective, open label, randomized, standard of care-controlled group (SOC) pilot trial compared inhaled oral and nasal administered BREATHOX^®^ (2.0 mg NaCl, particles size between 1–10 μm), with five or ten inhalations per day for ten days. The primary endpoint was the time to resolve COVID-19-related symptoms. Safety outcomes included adverse clinical and laboratory events. (3) Results: A total of 101 individuals were screened and 98 were randomly assigned to BREATHOX^®^ ten sessions per day (Group 1; 33 patients), BREATHOX^®^ five sessions per day (Group 2; 32 patients), or SOC (33 patients), and followed up for 28 days. There was an association with cough frequency after 10 days BREATHOX^®^ compared to SOC [Group 1: hazard ratio (HR) 2.01, 95% confidence interval (CI) 1.06–3.81; Group 2: HR 2.17, 95% CI 1.17–4.04]. No differences between the groups for the reported symptoms’ resolution time were seen after 28 days. After combining both BREATHOX^®^ groups, the period to cough resolution 10 days after randomization was significantly lower than in SOC (HR 2.10, 95% CI 1.20–3.67). An adverse event occurred in 30% of Group 1, 36% of Group 2, and 9% in SOC individuals. One patient from SOC had a serious adverse event. Nasal burning, sore or itchy nose, and dry mouth were considered related to BREATHOX^®^ use and resolved after stopping inhalations. (4) Conclusion: BREATHOX^®^ inhalation is safe and may be effective in reducing the duration of COVID-19-induced coughing.

## 1. Introduction

The first cases of coronavirus disease-2019 (COVID-19) were reported in December 2019. It was caused by the new severe acute respiratory syndrome coronavirus 2 (SARS-CoV-2). By the first weeks of January 2023, there had been approximately 666.8 million cases, which had varied from mild to very serious symptoms with 6.7 million deaths worldwide reported in the same period [1]. The most frequent symptoms in the mild disease were fever and cough [2,3,4,5].

Several anti-inflammatory treatments were investigated during the pandemic; they included cytokine modulation drugs to control the adverse effects of COVID-19. However, current guidelines for the mild disease recommend specific intervention to modify the life cycle of the virus in patients at high risk of disease progression [6].

The virus adheres to the mucosa of the upper respiratory epithelium and enters the target cell [7,8]. After replication in the nucleus, the virus is released from the cell by budding. This process occurs with the greatest intensity in Type I and II respiratory epithelial cells. The new viral particles have easy access to the bloodstream and become capable of infecting any other susceptible cells, such as cells of the kidney, heart, skeletal muscle, and endocrine glands, providing the peak of viremia [7,8,9,10].

A previous in vitro study showed an 88% inhibition of virus replication in SARS-CoV-2-infected monkey lung cells exposed to a 1.1% concentration hypertonic saline solution [11]. The inhibition mechanism is not known; one hypothesis is that the hyperosmotic environment triggered by the high extracellular sodium concentration changes the function of different membrane channels and prevents or attenuates virus entry into the cell. Another hypothesis is that the higher salinity increases activity of membrane Na^+^/K^+^ channels, leading to increased energy expenditure, and thereby reduced energy availability for virus replication [11,12].

Thus, we hypothesized that the use of inhaled NaCl particles, through BREATHOX^®^, would reduce respiratory symptoms in patients with mild COVID-19. Therefore, the primary aim of this study was to evaluate the effects of oral and nasal inhalation of hypertonic NaCl particles (BREATHOX^®^) on the duration of COVID-19-induced acute symptoms compared to the standard of care (SOC). Secondarily, we analyzed the occurrence of adverse events related to the device use.

## 2. Materials and Methods

### 2.1. Study Design

The BREATH study was a prospective, open label, randomized, pilot trial using inhaled oral and nasally administered BREATHOX^®^ (2.0 mg of NaCl—particle sizes 1–10 μm) for ten (Group 1) or five (Group 2) sessions per day for ten days. The results were compared to individuals subjected to SOC, antipyretic and/or analgesic, (Group 3) at a 1:1:1 ratio. The trial protocol was designed by the study steering committee.

### 2.2. Trial Population

#### 2.2.1. Inclusion Criteria

- Positive COVID-19 test by PCR test based on nasopharyngeal swab specimen or antigen swab test performed at the local laboratory;
- Patients above 18 years old;
- A diagnosis of mild COVID-19 within 24 h of enrolment with at least one infection symptom: (i) fever for more than 24 h, (ii) headache, (iii) sore throat, (iv) nasal obstruction, (v) cough, (vi) fatigue, (vii) chest pain, (viii) dyspnea, (ix) myalgia, (x) anosmia, (xi) ageusia, or (xii) gastrointestinal changes.

#### 2.2.2. Exclusion Criteria

- Pulse oximetry arterial saturation (SpO2) < 92%;
- Tachypnoea (respiratory rate ≥ 30 breaths/min);
- Current treatment in-hospital;
- Immediate consideration for hospital admission;
- Positive pregnancy test in women of childbearing age;
- Lactating women;
- Patients with unstable chronic disease and organ failure, judged by physician discretion;
- Patients with asthma, chronic obstructive lung disease, or other chronic respiratory diseases;
- Use of inhaled, oral, or intravenous corticosteroid in the ten days prior to randomization;
- Previous treatment with hydroxychloroquine and ivermectin in the last 10 days;
- Use of any investigational or unregistered product within the previous 3-month or the 5-half-life period before baseline, whichever is longer;
- Baseline biochemistry showing hemoglobin < 9.0 g/dL, absolute neutrophil count ≤ 1000/mm^3^, platelets ≥ 100,000/mm^3^, or creatinine clearance ≤ 30 mL/min (using the Cockcroft–Gault formula).

### 2.3. Outcomes

The primary endpoint was the reported time to resolution of COVID-19-related symptoms within 10 and 28 days: fever, headache, sore throat, nasal obstruction, cough, fatigue, chest pain, dyspnea, myalgia, anosmia, dysgeusia, or gastrointestinal changes.

### 2.4. Safety

Safety outcomes included adverse clinical and laboratory events.

### 2.5. Trial Procedures

Randomization was performed through an online web-based system (REDCap^®^) using computer-generated random numbers and was age-stratified to allow equal numbers of patients > 40 years old in each group. The randomization procedure was blinded to the investigators. Data was entered directly into REDCap by the study staff. Physicians, patients, and individuals who assessed outcomes were not blinded for the assigned treatment. The REDcap system was also used as the electronic case report form (eCRF).

### 2.6. Clinical and Laboratory Data

Data on the demographic characteristics and physiological variables were collected. Participants were requested to report their health status according to the Ordinal Scale for Clinical Improvement [13]. Laboratory analyses (complete blood count, urea, and creatinine) were performed at baseline or when necessary for complementary assessment in unscheduled visits.

### 2.7. Intervention

Group 1 was instructed to use BREATHOX^®^ ten sessions per day (two oral inhalations and one nasal inhalation in each nostril, 2 mg per inhalation) at hourly intervals during the daytime. Group 2 was instructed to use BREATHOX^®^ five sessions per day (two oral inhalations and one nasal inhalation in each nostril, 2 mg per inhalation), at three-hour intervals during the daytime. The treatment period was 10 days. Protocol adherence related to the medication use was assessed daily until day 10. Protocol adherence was considered acceptable at 80%. The SOC group was allowed to use antipyretics and/or analgesics; Groups 1 and 2 were allowed to use SOC at their own discretion.

All participants completed a daily symptom diary. Patients remained in the study for 28 days. The number of days with symptoms was counted from the day of inclusion. Phone interviews were carried out on day 10, and on-site visit or phone interviews were carried out on day 28. During the interviews, symptom evaluation and occurrence of endpoints or adverse events were evaluated.

### 2.8. Statistical Analysis

As a pilot study, we decided to include a study population of 100 patients. Statistical analysis was performed by intention to treat (ITT). Therefore, all randomized patients were analyzed according to their allocated group, regardless of adherence to treatment. The per protocol (PP) population consisted of all subjects with good compliance and without a major protocol deviation (including but not limited to violation of inclusion and exclusion criteria). The PP and ITT analyses were used to evaluate the primary endpoints. All subjects who received at least one dose of BREATHOX^®^ or were included in the SOC group made up the safety analysis set.

Descriptive statistics for the endpoints are presented according to the treatment group and number of visits (if applicable). Continuous data are presented as the number of subjects and reported as mean and standard deviation (SD). The mean symptom resolution time reported was calculated for each group. The safety data are presented using descriptive statistics. Kaplan–Meier curves were used to demonstrate symptom resolution time. Proportional Cox regression models were performed and a *p*-value of ≤ 0.003 was considered statistically significant for multiple comparisons adjusted for the Bonferroni method. The test considered the null hypothesis as the outcome being the same in all groups. The alternate hypothesis means at least one pair of groups is different. Statistical analyses were carried out using Stata Software, version 16.0 (Statcorp, College Station, TX, USA). Statistical oversight and analyses were performed at Pelotas Federal University, Brazil.

## 3. Results

**Figure 1:**
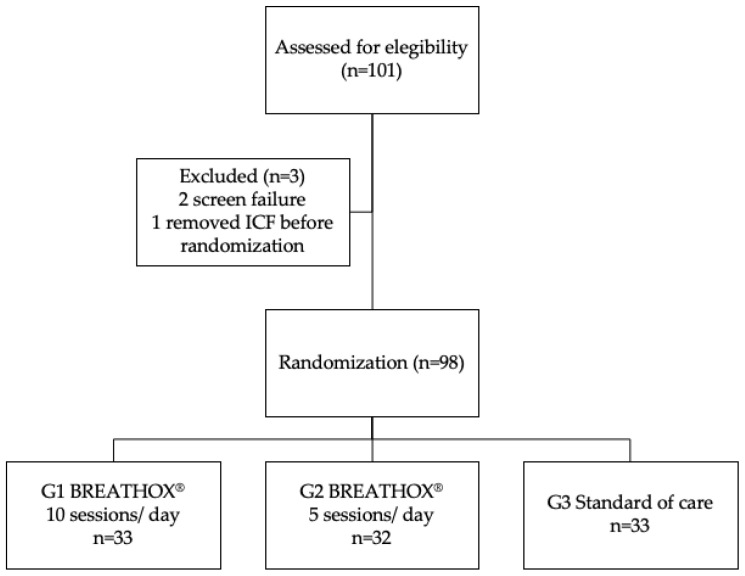
Flow diagram.

One hundred and one individuals were screened, and 98 patients were included from 1 December 2021, to 3 March 2022. In total, 33 patients were assigned to Group 1, 32 patients to Group 2, and 33 to SOC. Primary endpoints were available for all individuals (Figure 1).

**Figure 2:**
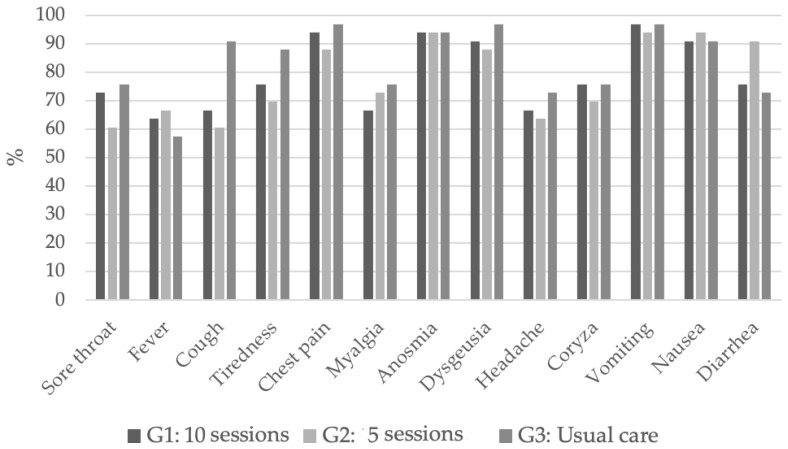
Distribution of COVID-19 symptoms at baseline. G1: Group 1, BREATHOX^®^ 10 sessions/day; G2: Group 2, BREATHOX^®^ 5 sessions/day; G3: Group 3, SOC.

Baseline data are shown in Table 1. Eighty-eight individuals had had a complete COVID-19 vaccination scheme (62.5% AZD1222 (AstraZeneca, Oxford, UK) and 37.5% Coronavac (Sinovac Biotech, Beijing, China)), and 82 patients had received one COVID-19 booster vaccine (81.0% Pfizer–BioNTech COVID-19 vaccine; 13.4% AstraZeneca; 4.6% Coronavac). The baseline COVID-19 symptoms frequency is shown in Figure 2. In contrast to SOC, both Groups 1 and 2 exhibited a diminished proportion of study participants who continued to experience coughing (*p* = 0.014); however, the prevalence of other symptoms remained consistent across all groups. The percentage of patients who used BREATHOX^®^ according to the prescription was 69.2% in Group 1 and 73.3% in Group 2.

Cox regression models showed a statistically significant difference in cough frequency after 10 days BREATHOX^®^ use in both groups when compared to SOC [Group 1: hazard ratio (HR) 2.01, 95% confidence interval (CI) 1.06–3.81; Group 2: HR 2.17, 95% CI 1.17–4.04]. No differences in symptom frequencies were seen after 28 days between the groups. The hazard ratios for all symptoms are summarized in Table 2.

As a secondary analysis, we combined both BREATHOX^®^ groups and compared them to the SOC group. Cough resolution 10 days after randomization was significantly lower in the BREATHOX^®^ groups than SOC (HR 2.10; 95% CI 1.20–3.67) (Table 3).

One serious adverse event was registered: one patient from the SOC group was hospitalized with unstable angina. An external independent adjudication evaluation considered it unrelated to the study intervention, but related to the SARS-CoV-2 infection. The proportion of any adverse event was 30% in Group 1, 36% in Group 2, and 9% in the SOC group. Adverse effects are presented in Table 4. Nasal burning, a sore or itchy nose, and a dry mouth were considered related to BREATHOX^®^ use and were resolved after stopping BREATHOX^®^ inhalation.

## 4. Discussion

This study investigated the effects of a new intervention aimed at controlling respiratory symptoms in mild COVID-19. We showed that the period to reported cough resolution was lower in both BREATHOX^®^ groups than the SOC group. The effect of BREATHOX^®^ on coughing may hypothetically be related to a hyperosmotic response influencing the function of different membrane channels consequently preventing virus entry into the cells. Moreover, the hypertonic solution may increase the mucociliary clearance and reduce the destructive inflammatory process in the airways with a decrease in respiratory symptoms. Hypertonic saline solution has been widely used in patients with acute respiratory diseases as an adjuvant to other therapies for improving respiratory symptoms [14,15,16,17].

In the three years of the COVID-19 pandemic, scientists have developed strategies to recommend the best evidence for treating SARS-CoV-2 infections. Pharmacological guidelines are prepared in accordance with the disease severity [6,18]. The main goal of intervention is to reduce the risk of death and the need for mechanical ventilation in moderate–severe COVID-19 cases. Therefore, the use of antiviral drugs, corticosteroids, and immunosuppressive interventions is recommended for moderate to severe COVID-19 cases [6,18]. Indeed, monoclonal antibodies are not recommended in SARS-CoV-2 pre-exposure or to control the progression of disease since the omicron sub-lineages escape the monoclonal antibodies [6].

Most of the pharmacological guidelines for COVID-19 treatment were designed for previous sub-lineages of SARS-CoV-2, such as Alpha, Beta, Gama, and Delta which cause lower respiratory tract infections [1]. Currently, the Omicron variant represents the most divergent mutation across the world, which has modified the disease approach due to its higher transmission rate and milder clinical picture. Most of the patients with the Omicron variant present a mild illness with fever, cough, sore throat, malaise, headache, muscle pain, ageusia, and anosmia. Therefore, treatment is conducted at home and often through telemedicine. In this case, antiviral drugs are recommended to specific populations with a high probability of developing severe COVID-19. On the other hand, patients without comorbidities or risk factors may use symptomatic treatment to relieve symptoms. However, the use of drugs commonly prescribed for the common cold may not relieve symptoms and BREATHOX^®^ can be considered in this population.

Mild COVID-19 may be associated with mild respiratory symptoms such as coughing. NICE guidelines recommend the use of non-pharmacological treatments as the first line, such as honey, to manage COVID-19-induced coughing. This should be followed by codeine linctus, codeine phosphate, or morphine sulfate [18]. In this randomized controlled trial, we showed positive results for cough resolution with BREATHOX^®^ in mild COVID-19.

Concerning the administration method, in addition to direct application in the upper airways, the inhalation of hypertonic saline has been shown to improve quality of life for COPD patients. Therefore, the availability of inhaler and nasal devices with hypertonic saline may facilitate the care of patients with chronic respiratory diseases [16,17]. We did not observe an increased adverse event severity related to BREATHOX^®^ use; most of the mild adverse events were nasal burning which did not require modification of treatment. This demonstrates the opportunity to conduct a novel study on coughing caused by mild COVID-19 by using a symptomatic relief intervention.

There are limitations to this study. As a pilot trial, it is possible that the low sample size prevented statistical differences from being found between the BREATHOX^®^ and SOC groups in COVID-19-related symptoms other than coughing. Additionally, we did not conduct an analysis of the virus cycle threshold using BREATHOX^®^, which represents a gap in our investigation. Large double-blinded, placebo-controlled randomized clinical trials are therefore needed to confirm the efficacy of BREATHOX^®^ on cough resolution following COVID-19 and to further investigate BREATHOX^®^ efficacy on other COVID-19 symptoms. Also, the results may be biased by the lack of a placebo-controlled group.

## 5. Conclusions

The inhaled use of BREATHOX^®^ was safe and effective in reducing the duration of COVID-19-induced coughing in the studied population.

## Figures and Tables

**Table 1:** Patient demographics and baseline clinical data.

	G1	G2	G3	*p*
Age, years (mean, IIQ)	41 (33–47)	44.5 (31–51.3)	40 (28–48)	0.672
Sex (female, %)	63.6	60.6	60.6	0.958
Skin color (white, %)	78.8	63.6	69.7	0.409
Comorbidities (%)	24.2	27.3	24.2	0.948
Type 1 diabetes (%)	0	0	3.03	0.364
Diabetes mellitus (%)	3.03	6.06	9.09	0.587
Obesity (%)	15.2	15.2	18.2	0.928
Arterial hypertension (%)	15.2	21.1	15.2	0.753
Coronary artery disease (%)	0	3.03	6.1	0.357
HIV (%)	0	0	3.03	0.364
Auto-immune disease (%)	12.1	6.06	6.06	0.580
Allergy (%)	12.1	15.2	15.2	0.920

G1: Group 1, BREATHOX^®^ 10 sessions/day; G2: Group 2, BREATHOX^®^ 5 sessions/day; G3: Group 3, standard of care; SD: standard deviation; HIV: human immunodeficiency virus infection; IIR: interquartile range.

**Table 2:** Time to reported symptoms resolution in comparison to the SOC group.

	Day 10					Day 28				
Outcomes Symptoms	G1		G2			G1		G2		
	HR	95% CI	HR	95% CI	*p*-Value	HR	95% CI	HR	95% CI	*p*-Value
Sore throat	1.39	0.80–2.42	1.63	0.92–2.91	0.234	1.04	0.61–1.78	1.45	0.84–2.51	0.365
Fever	0.96	0.57–1.62	0.87	0.51–1.47	0.895	0.83	0.49–1.40	0.82	0.48–1.38	0.700
Cough	2.17	1.17–4.04	2.01	1.06–3.81	0.034	1.64	0.96–2.81	1.46	0.86–2.50	0.163
Tiredness	0.99	0.55–1.76	1.16	0.65–2.06	0.831	0.87	0.52–1.47	0.99	0.58–1.67	0.862
Chest pain	0.98	0.59–1.62	0.93	0.55–1.57	0.963	0.86	0.52–1.42	0.87	0.52–1.46	0.805
Myalgia	1.24	0.72–2.14	1.06	0.60–1.89	0.729	0.96	0.56–1.65	0.90	0.53–1.56	0.936
Anosmia	0.97	0.57–1.65	0.88	0.51–1.51	0.897	0.85	0.51–1.43	0.84	0.50–1.41	0.759
Dysgeusia	1.05	0.62–1.77	1.00	0.59–1.69	0.980	0.89	0.53–1.49	0.89	0.53–1.48	0.871
Headache	1.33	0.77–2.29	1.01	0.58–1.76	0.506	1.17	0.67–2.03	1.07	0.63–1.82	0.859
Coryza	0.95	0.54–1.67	0.65	0.36–1.19	0.340	0.90	0.52–1.55	0.72	0.42–1.24	0.487
Vomiting	1.00	0.60–1.66	0.96	0.58–1.61	0.988	0.87	0.52–1.45	0.90	0.54–1.50	0.857
Nausea	1.05	0.63–1.76	1.02	0.61–1.72	0.980	0.92	0.55–1.54	0.96	0.58–1.59	0.948
Diarrhea	1.03	0.61–1.73	0.99	0.59–1.68	0.990	0.89	0.53–1.50	0.93	0.55–1.55	0.911

G1: Group 1, BREATHOX^®^ 10 sessions/day; G2: Group 2, BREATHOX^®^ 5 sessions/day; HR: hazard ratio; CI: 95% confidence interval for each group compared to the SOC group. Note 1: *p*-values refer to the overall comparisons, being the null hypothesis that all groups are equal. Note 2: due to the multiple comparisons performed, the corrected threshold for the *p*-value adjusted for Bonferroni method was 0.003.

**Table 3:** Time to reported symptoms resolution in combined Groups 1 and 2 compared to SOC group.

	Day 10			Day 28		
Symptom	HR	95% CI	*p*-Value	HR	95% CI	*p*-Value
Sore throat	1.49	0.91–2.44	0.111	1.20	0.76–1.91	0.433
Fever	0.91	0.58–1.44	0.700	0.82	0.53–1.29	0.399
Cough	2.10	1.20–3.67	0.010	1.54	0.98–2.45	0.063
Tiredness	1.07	0.65–1.76	0.790	0.93	0.59–1.44	0.732
Chest pain	0.96	0.62–1.48	0.839	0.86	0.56–1.34	0.513
Myalgia	1.15	0.71–1.87	0.565	0.93	0.59–1.48	0.763
Anosmia	0.92	0.58–1.47	0.736	0.85	0.55–1.31	0.458
Dysgeusia	1.02	0.65–1.61	0.930	0.89	0.57–1.38	0.599
Headache	1.16	0.72–1.86	0.545	1.11	0.70–1.77	0.649
Coryza	0.79	0.48–1.30	0.360	0.80	0.50–1.26	0.333
Vomiting	0.98	0.6–1.53	0.931	0.89	0.57–1.37	0.590
Nausea	1.04	0.66–1.62	0.867	0.94	0.60–1.46	0.775
Earache	1.00	0.65–1.54	0.989	0.90	0.59–1.39	0.647
Skin rash	1.00	0.65–1.54	0.989	0.90	0.59–1.39	0.647
Diarrhea	1.01	0.64–1.59	0.960	0.91	0.58–1.42	0.682

Group 1: BREATHOX**^®^** 10 sessions/day; Group 2: BREATHOX**^®^** 5 sessions/day; HR: hazard ratio; CI: 95% confidence interval for each interval compared to SOC group. Note: due to the multiple comparisons performed, the corrected threshold for the *p*-value adjusted for Bonferroni method was 0.003.

**Table 4:** Number of adverse events.

	G1	G2	G3
Blood pressure increase	0	1	0
Neck pain	1	0	0
Cystitis	0	1	0
Dyslipidemia	0	0	1
Dyspnea	1	0	0
Ankle pain	0	1	0
Sneezing	0	1	0
Pharyngitis	0	0	2
Nausea	1	0	0
Bacterial rhinosinusitis	0	1	0
Dry mouth	0	1	0
Nasal burning	6	9	0
Chest pain	0	1	1
Sore or itchy nose	1	1	0

G1: Group 1, BREATHOX^®^ 10 sessions/day; G2: Group 2, BREATHOX^®^ 5 sessions/day; G3: Group 3, SOC.

## Data Availability

The datasets used and/or analyzed during the current study are available from the corresponding author on reasonable request.

## References

[1] WHO (2020). WHO COVID-19 Dashboard.

[2] Tay M.Z., Poh C.M., Rénia L., MacAry P.A., Ng L.F.P. (2020). The trinity of COVID-19: Immunity, inflammation and intervention. Nat. Rev. Immunol..

[3] Guan W., Ni Z.Y., Hu Y., Liang W.H., Ou C.Q., He J.X., Liu L., Shan H., Lei C.L., Hui D.S.C. (2020). Clinical Characteristics of Coronavirus Disease 2019 in China. N. Engl. J. Med..

[4] Chen Y., Geng Y., Xu X., Chen X., Gao J., Li J., Zhang X. (2021). The features comparison between patients in the ICU and general wards and between patients with different outcomes: A 2020 COVID-19 study. Ann. Palliat. Med..

[5] Huang C., Wang Y., Li X., Ren L., Zhao J., Hu Y., Zhang L., Fan G., Xu J., Gu X. (2020). Clinical features of patients infected with 2019 novel coronavirus in Wuhan, China. Lancet.

[6] COVID-19 Treatment Guidelines Panel (2022). Coronavirus Disease 2019 (COVID-19) Treatment Guidelines.

[7] Brito S.B.P., Braga I.O., Cunha C.C., Takenami I. (2020). Mecanismos imunopatológicos envolvidos na infecção por SARS-CoV-2. J. Bras. Patol. Med. Lab..

[8] Jin Y.H., Cai L., Cheng Z.-S., Cheng H., Deng T., Fan Y.-P., Fang C., Huang D., Huang L.-Q., Huang Q. (2020). A rapid advice guideline for the diagnosis and treatment of 2019 novel coronavirus (2019-nCoV) infected pneumonia (standard version). Mil. Med. Res..

[9] Li X., Geng M., Peng Y., Meng L., Lu S. (2020). Molecular immune pathogenesis and diagnosis of COVID-19. J. Pharm. Anal..

[10] Wang X., Ding Y.Q. (2020). From SARS to COVID-19: Pathogens, receptor, pathogenesis and principles of the treatment. Zhonghua Bing Li Xue Za Zhi.

[11] Machado R.R.G., Glaser T., Araujo D.B., Petiz L.L., Oliveira D.B.L., Durigon G.S., Leal A.L., Pinho J.R.R., Ferreira L.C.S., Ulrich H. (2021). Inhibition of Severe Acute Respiratory Syndrome Coronavirus 2 Replication by Hypertonic Saline Solution in Lung and Kidney Epithelial Cells. ACS Pharmacol. Transl. Sci..

[12] Silva De Souza A., Rivera J.D., Almeida V.M., Ge P., de Souza R.F., Farah C.S., Ulrich H., Marana S.R., Salinas R.K., Guzzo C.R. (2020). Molecular Dynamics Reveals Complex Compensatory Effects of Ionic Strength on the Severe Acute Respiratory Syndrome Coronavirus 2 Spike/Human Angiotensin-Converting Enzyme 2 Interaction. J. Phys. Chem. Lett..

[13] World Health Organization (2020). WHO R&D Blueprint Novel Coronavirus COVID-19 Therapeutic Trial Synopsis.

[14] Schoeffei R.E., Anderson S.D., Altounyan R.E.C. (1981). Bronchial hyperreactivity in response to inhalation of ultrasonically nebulised solutions of distilled water and saline. Br. Med. J..

[15] Wark P., Mcdonald V.M. (2018). Nebulised hypertonic saline for cystic fibrosis. Cochrane Database Syst. Rev..

[16] Taube C., Holz O., Mücke M., Jorres R.A., Magnussen H. (2001). Airway response to inhaled hypertonic saline in patients with moderate to severe chronic obstructive pulmonary disease. Am. J. Respir. Crit. Care Med..

[17] Rytilä P.H., Lindqvist A.E., Laitinen L.A. (2000). Safety of sputum induction in chronic obstructive pulmonary disease. Eur. Respir. J..

[18] COVID-19 Rapid Guideline: Managing COVID-19—The National Institute for Health and Care Excellence (NICE). 28.0 published on 29.03.2023. https://www.nice.org.uk/guidance/ng191.

